# Prolonged Extreme Cold Water Diving and the Acute Stress Response During Military Dive Training

**DOI:** 10.3389/fphys.2022.842612

**Published:** 2022-07-08

**Authors:** Karen R. Kelly, Laura J. Arrington, Jake R. Bernards, Andrew E. Jensen

**Affiliations:** ^1^ Applied Translational Exercise and Metabolic Physiology Team, Warfighter Performance, Naval Health Research Center, San Diego, CA, United States; ^2^ Leidos, Inc., San Diego, CA, United States

**Keywords:** cortisol, osteocalcin, alpha-amylase, thermoregulation, arctic conditions

## Abstract

**Introduction:** Cold water exposure poses a unique physiological challenge to the human body. Normally, water submersion increases activation of parasympathetic tone to induce bradycardia in order to compensate for hemodynamic shifts and reduce oxygen consumption by peripheral tissues. However, elevated stress, such as that which may occur due to prolonged cold exposure, may shift the sympatho-vagal balance towards sympathetic activation which may potentially negate the dive reflex and impact thermoregulation.

**Objective:** To quantify the acute stress response during prolonged extreme cold water diving and to determine the influence of acute stress on thermoregulation.

**Materials and Methods:** Twenty-one (*n* = 21) subjects tasked with cold water dive training participated. Divers donned standard diving equipment and fully submerged to a depth of ≈20 feet, in a pool chilled to 4°C, for a 9-h training exercise. Pre- and post-training measures included: core and skin temperature; salivary alpha amylase (AA), cortisol (CORT), osteocalcin (OCN), testosterone (TEST) and dehydroepiandosterone (DHEA); body weight; blood glucose, lactate, and ketones.

**Results:** Core, skin, and extremity temperature decreased (*p* < 0.001) over the 9-h dive; however, core temperature was maintained above the clinical threshold for hypothermia and was not correlated to body size (*p* = 0.595). There was a significant increase in AA (*p* < 0.001) and OCN (*p* = 0.021) and a significant decrease in TEST (*p* = 0.003) over the duration of the dive. An indirect correlation between changes in cortisol concentrations and changes in foot temperature (*ρ* = -0.5,*p* = 0.042) were observed. There was a significant positive correlation between baseline OCN and change in hand temperature (*ρ* = 0.66, *p* = 0.044) and significant indirect correlation between changes in OCN concentrations and changes in hand temperature (*ρ* = -0.59, *p* = 0.043).

**Conclusion:** These data suggest that long-duration, cold water diving initiates a stress response—as measurable by salivary stress biomarkers—and that peripheral skin temperature decreases over the course of these dives. Cumulatively, these data suggest that there is a relationship between the acute stress response and peripheral thermoregulation.

## Introduction

Divers are exposed to the extreme environment of the open sea, including extreme pressure and temperature variability—. Perhaps the most inescapable environmental factor that affects military divers is cold exposure. The initial physiological and thermoregulatory response to cold exposure, known as the cold shock response (CSR), causes a rapid decrease in skin temperature which leads to veno- and vaso-constriction, and is accompanied by a decrease in peripheral blood volume/flow (Tipton, 1989). The rapid change in blood flow distribution is attributed to *α*-adrenergic receptor activation in response to decreased skin temperature and elevated norepinephrine concentrations (Sramek et al., 2000). The increase in norepinephrine from CSR and the stress of operations/training results in an increased sympathetic drive along with an increased heart rate (Castellani and Young, 2016). Additionally, cold water poses a unique physiological challenge to the human body. Normally, water submersion increases activation of parasympathetic tone to induce bradycardia in order to compensate for hemodynamic shifts and reduce oxygen consumption by peripheral tissues (i.e., mammalian dive reflex (Godek and Freeman, 2021). However, elevated stress, such as that which may occur due to prolonged cold exposure, may shift the sympatho-vagal balance towards sympathetic activation (Schipke and Pelzer, 2001) potentially negating the dive reflex (Godek and Freeman, 2021) and altering thermoregulatory response at the extremities.

Physiological stress (such as that endured in extreme cold environments), physical stress, and psychological stress all elicit a nearly identical physiological (stress) response (Morgan, et al., 2000a; Castellani et al., 2002; Choshen-Hillel et al., 2021). This physiological stress response is primarily propagated by two neuroendocrine systems: the sympathetic-adreno-medullary (SAM) axis/sympathetic nervous system (SNS) and the hypothalamic-pituitary-adrenal (HPA) axis. Both, SAM/SNS and HPA axis, operate in a concerted effort to maintain homeostasis and promote host survival (De Kloet et al., 1998; Chen et al., 2017). As its name implies, the SAM/SNS is directly responsible for changes in sympatho-vagal balance and is supported by downstream alterations in the HPA axis. A typical stress response results in immediate SAM/SNS activation; whereas, the HPA axis shows a somewhat slower response (Fragala et al., 2011). A downstream biomarker of the HPA axis, cortisol, has multiple roles in the body to support the “fight-or-flight” response of the sympathetic nervous system (Taylor et al., 2016). Under normal stressful circumstances, cortisol is secreted in response to an increase in sympathetic drive (Taylor et al., 2016); however, diving induces parasympathetic dominance in order to accommodate for blood volume shifts due to the undersea environment (Chouchou et al., 2009; Noh et al., 2018). The effect of this potential “push-pull” between parasympathetic and sympathetic dominance is unexamined in divers. Limited studies in recreational divers have focused on the cortisol response to changes in environmental pressure (depth) during short duration (15–20 min) dives; yet the impact on thermoregulation has not been examined (Weist et al., 2012; Zarezadeh and Azarbayjani, 2014).

To date, there are limited data on thermoregulation during prolonged arctic-like dives in wetsuits (Lundell et al., 2019). Recently, we demonstrated that closed cell wetsuits effectively thermoregulate during a 6-h dive in 5°C and that was independent of body size or composition (Chapin et al., 2021). Further, there was a 53% increase in metabolic rate in response to the stress of prolonged cold exposure and to effectively maintain core temperature. Furthermore, peripheral skin and extremity temperatures declined but remained above 10°C; the threshold for non-freezing cold injury (Imray et al., 2011). While these physiological adjustments were critical for survival, quantification of the stress load is unknown. As military divers continue to train and operate in arctic-like conditions; the use of wetsuits for mobility versus class dry suits is more desirable. As such, the need to understand the pathophysiological adjustments to maintain homeostatsis is critical. Also, understanding the allostatic load on a military diver will provide documentation of health and habituation to the stress (McEwen, 2015).

Assessments of the neuroendocrine biochemicals involved in stress pathways systems can objectively quantify the stress level observed. In fact, easily captured non-invasive salivary measures of alpha-amylase (AA), cortisol (CORT) and dehydroepiandosterone (DHEA)—which represent the SAM/SNS and HPA axis—can be used to quantify stress levels. Capturing these measures along with salivary osteocalcin (OCN), which has also been shown to respond to stress and modulate glucose metabolism and testosterone (TEST), may provide a clear picture of the acute stress response during cold water diving. Thus, the primary purpose of this effort is to quantify the acute stress response during prolonged extreme cold water diving and determine the influence on thermoregulation. We hypothesize that individuals that reach thermoregulatory thresholds of hypothermia and non-freezing cold injury will have an exaggerated stress response. A secondary goal was to observe the cold tolerance limits and duration of submersion in neoprene wetsuits to determine the threshold of thermoregulation.

## Material and Methods

### Ethics Statement

This study was approved by the Institutional Review Board at Naval Health Research Center and adhered to Department of the Navy human research protection policies (Protocol NHRC. 2020.0004). All participants provided written informed consent.

#### Participants

Twenty-one male military divers who were tasked with cold water submersion, were recruited, and participated in this study. The divers were categorized into two participant groups (PG) based upon tasks that they were responsible for. Participant group one (PG1, *n* = 11) are support divers and PG2 are submersible vehicle divers (*n* = 10).

#### Scenario

A pool and a refrigerated truck were chilled to approximately 4°C and 0°C, respectively. There were two different scenarios occurring concurrently to reflect occupational tasks. PG1 divers were responsible for launching a submersible and maintaining a dive depth at approximately 0.605 atm (atm) for 9 h. The submersible completed a mixed environment scenario and were divided into two groups (PG2A, *n* = 4 and PG2B, *n* = 6). PG2A managed the submersible at 0.302 atmwhile PG2B transitioned between water and land. Durations of time in the water and on land (in a freezer truck) were as follows: PG2A and PG2B collectively started the scenario with a 1.5-h dive at 10 fsw, from hour 1.5 to 6.5 of evalution, PG2B transitioned to the freezer truck (land) while PG2A remained in the water submerged. At hour 6.5 of training, PG2B returned to the water and both PG2B and PG2A submerged from hour 6.5 to nine upon which time the scenario was terminated. While submerged both PG1 and PG2A were passive with no movement. PG2B briefly walked from the pool to the freezer truck located approximately 15 m away. While in the truck, PG2B sat quietly for 5 h with no movement beyond occasionally standing to switch positions.

#### Thermoregulation

The night before the dive, single use ingestible core temperature monitoring pills (Vital Sense, Phillips Respironics, Bend, Oregon), were activated and distributed. Divers were instructed to ingest the pill on the morning of the dive at 0500, approximately 4 h prior to dive splash to allow for ample transit time to the lower gastrointestinal tract. Thermistor-based capsules have a sensing range of 25°C to 50°C with reported accuracy ±0.1°C. Capsules pass through the GI tract without affecting bodily functions and are easily passed. Immediately prior to dive, skin temperature patches (Vital Sense, Philips Respironics, Bend, Oregon) were activated and affixed to the right side of the participant at the following sites: dorsal hand, dorsal foot, mid pectoralis (chest), lateral deltoid (arm), mid-thigh (thigh), and mid-calf (leg). Mean skin temperature was calculated using four of the six sites; chest, arm, thigh, and leg (Ramanathan, 1964). Skin and core temperature readings were collected every 30 min throughout the 6-h and 9-h training evolutions. Termination criteria was 10°C skin temperature or 35°C core temperature. Risk of non-freezing cold injury occurs at 10°C and as such, served as the termination criteria for safety mitigation of nerve damage (Imray et al., 2011).

Mean skin temperature (Ramanathan, 1964):

$$T_{sk}=0.3\left(T_{chest}+T_{arm}\right)+0.2\left(T_{thigh}+T_{leg}\right)$$

#### Diving equipment

Participants wore thermoprotective gear which consisted of a closed cell long farmer john wetsuit bottom, a long sleeve top, booties, and gloves to their personal preference Overall thickness at torso was 17.5 mm, legs and arms 10 mm, feet 10 mm, and hands 10 mm. Breathing gas was supplied to divers using self-contained underwater breathing apparatus (SCUBA) closed-circuit oxygen rebreathers to eliminate bubbles. The apparatus provides 100% oxygen to the diver, recycling expelled breath into a closed circuit where it is filtered for carbon-dioxide.

#### Physiological metrics

Pre- and post-dive, body weight, urine specific gravity, blood glucose, lactate, and ketones were measured. Weight was measured using a digital scale calibrated to the nearest 0.01 kg (SECA, Germany). Height was measured to the nearest 0.01 cm using a stadiometer (SECA, Germany). Heart rate was continuously measured throughout the dive using Polar Team Pro Sensors (Polar Electro, Bethpage, New York). Prior to the dive, individual profiles were created with individuals’ demographic information. Polar software provided estimated calorie expenditure data from predictive equations using collected data and participant information.

#### Stress Hormones

Saliva was collected for acute stress biomarkers immediately pre- and post-dive. For saliva collection, a small cotton swab will be placed in the mouth for 3 min until fully saturated with saliva, then placed in a conical vial, and then placed immediately on ice while transferred to a -20°C freezer. Collected samples were analysed in duplicate for stress and sex-steroid specific hormones to include Dehydroepiandrosterone (DHEA), total testosterone (TEST), cortisol (CORT), alpha-amylase (AA) and osteocalcin (OCN) *via* Enzyme-Linked Immunosorbent Assay (ELISA; Salimetrics LLC, State College, Pennsylvania).

### Statistical Analyses

All statistical analysis were performed using R version 4.1.0. Linear mixed models were employed to test the effect of dive time for divers. Within the model, dive time was included as the fixed effect while individuals were added as a random effect. Upon a statistically significant main effect of dive time, simple contrasts were performed comparing changes in temperatures at each hour to the initiation of the dive (splash). To limit the false discovery rate, simple contrasts were adjusted using the Benjamini–Hochberg procedure. Furthermore, changes in biomarkers pre and post dive were examined using the Wilcoxon signed-rank paired test. Lastly, Spearman rank correlation coefficients were calculated to examine the relationship between changes in thermoregulation and both baseline, and relative changes in the biomarkers collected.

## Results

Participant demographic and anthropometric data are to be found in Table 1. There was a significant decrease in blood glucose pre to post dive for divers (PG2A: *p* = 0.007; PG1: *p* = 0.008) and ketones for PG2A and PG2B (*p* < 0.001). Physiological metrics are presented in Table 2.

**TABLE 1:** Demographic data of divers.

Measure	PG1	PG2
Age, yrs	27.60 ± 0.97	30.58 ± 1.47
Height, cm	178.05 ± 0.83	179.49 ± 1.96
Body Weight, kg	84.32 ± 2.33	85.57 ± 1.86
BSA, m2	2.02 ± 0.04	2.05 ± 0.03

Data are presented as Mean ± Standard Error. BSA, Body surface area.

**TABLE 2:** Physiological dive data.

Measure	PG1 Pre	PG1 Post	PG2 Pre	PG2 Post
Body Weight, kg	85.1 ± 1.96	82.1 ± 2.71	84.27 ± 2.42	84.61 ± 2.85
USG	1.017 ± 0.001	1.018 ± 0.001	1.019 ± 0.002	1.013 ± 0.001
Blood Lactate, mmol/L	1.44 ± 0.24	1.46 ± 0.19	1.08 ± 0.19	1.11 ± 0.24
Blood Glucose, mg/dL	99.1 ± 4.21	83.75 ± 2.34*	101.3 ± 3.14	83.25 ± 4.69*
Blood Ketones, mmol/L	0.22 ± 0.031	0.58 ± 0.22	0.17 ± 0.02	0.38 ± 0.02*

Data are presented as Mean ± Standard Error. USG, Urine Specific Gravity. *p* < 0.05.

### Thermoregulation

#### Core Temperature

**FIGURE 1:**
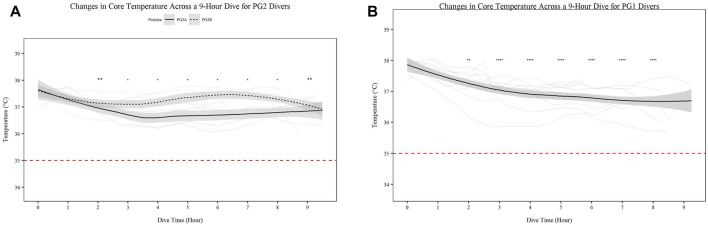
Changes in Core Temperature across a 9-h Dive for PG2A and PG2B **(A)** and PG1 **(B)** divers. Changes in core temperature for each diver across each dive are represented. Each diver is associated with a color found in the legend. PG2B are depicted as solid lines; and PG2A are depicted as dashed lines. The red dashed line represents the 35°C, the point of hypothermia onset. *p < 0.05, **p < 0.01, ***p < 0.001, ****p < 0.0001; * = PG2A, + = PG2B. All comparisons are in relation to Hour 0. Trend line created using loess function with standard error band. Red dashed line represents cutoff for hypothermia.

There was significant change in core temperature over time for all individuals (*p* < 0.001); however all divers remained above the 35°C threshold for hypothermia (Figures 1A,B).

#### Peripheral Temperature

**FIGURE 2:**
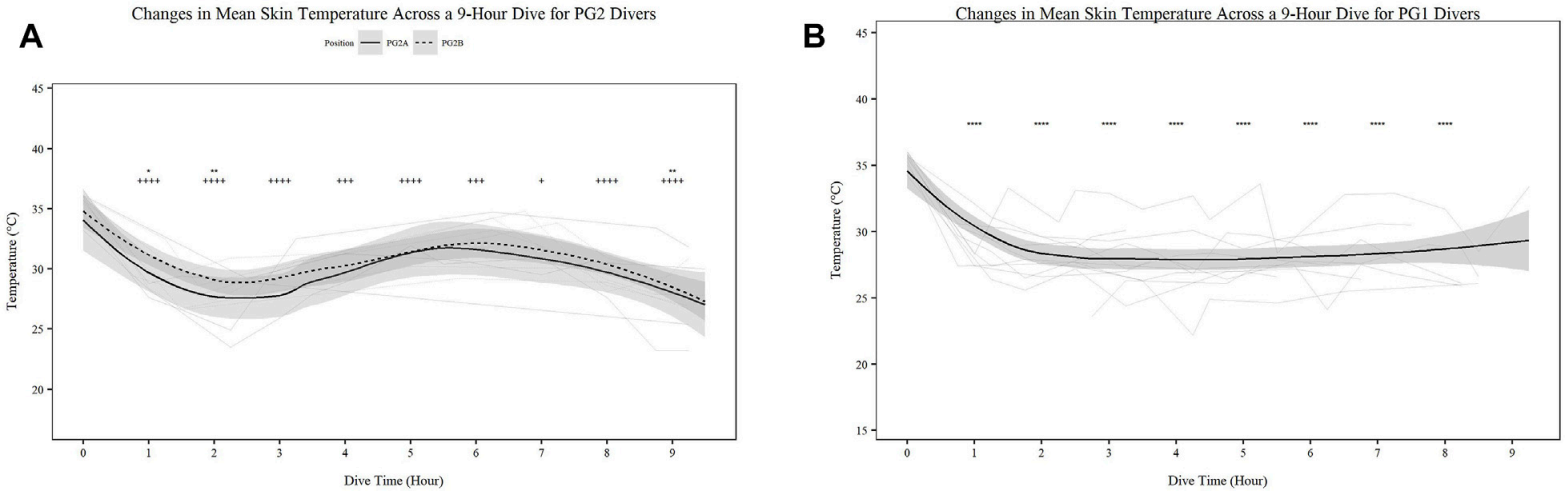
Changes in Mean Skin Temperature across a 9-h Dive for PG2A and PG2B **(A)** and PG1 **(B)** divers. Changes in skin temperature for each diver across each dive are represented. PG2B are depicted as solid lines; and pilots and navigators are depicted as dashed lines. The red dashed line represents the 10°C, the point of risk for non-freezing cold injuries. *p < 0.05, **p < 0.01, ***p < 0.001, ****p < 0.0001; * = PG2A, + = PG2B. All comparisons are in relation to Hour 0. Trend line created using loess function with standard error band.

**FIGURE 3:**
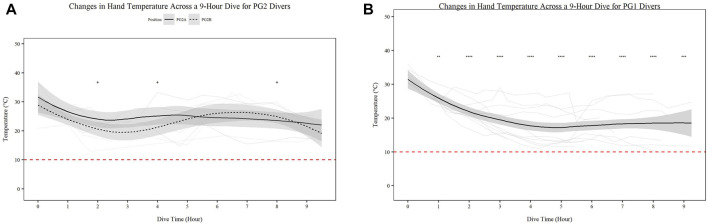
Changes in Hand Temperature across a 9-h Dive for PG2A and PG2B **(A)** and PG1 **(B)** divers. Changes in hand temperature for each diver across each dive are represented. PG2B are depicted as solid lines; and PG2A are depicted as dashed lines. The red dashed line represents the 10°C, the point of risk for non-freezing cold injuries. *p < 0.05, **p < 0.01, ***p < 0.001, ****p < 0.0001; * = PG2A, + = PG2B. All comparisons are in relation to Hour 0. Trend line created using loess function with standard error band.

**FIGURE 4:**
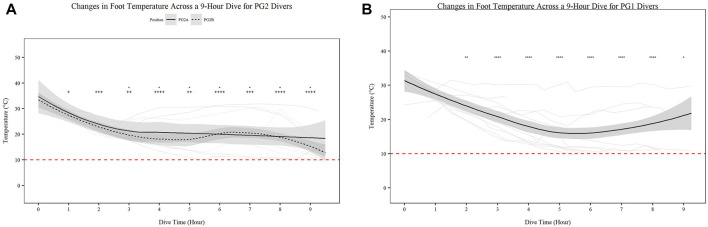
Changes in Foot Temperature across a 9-h Dive for PG2A and PG2B **(A)** and PG1 **(B)** divers. Changes in foot temperature for each diver across each dive are represented. PG2B are depicted as solid lines; and PG2A are depicted as dashed lines. The red dashed line represents the 10°C, the point of risk for non-freezing cold injuries. *p < 0.05, **p < 0.01, ***p < 0.001, ****p < 0.0001; * = PG2A, + = PG2B. All comparisons are in relation to Hour 0. Trend line created using loess function with standard error band.

Mean skin temperature (Figures 2A,B) significantly decreases for all participants over time (Figures 2A,B; *p* < 0.001). Further PG1 mean skin temperature decreased more so than PG2A and B collectively (*p* < 0.01). Hand (Figures 3A,B) and foot (Figures 4A,B) temperatures significantly decreased (*p* < 0.001) over time. Risk of non-freezing cold injury occurs at 10°C and as such, this temperature serves as the termination criteria for safety mitigation of nerve damage.

### Stress Response

#### Hormone Concentrations

**FIGURE 5:**
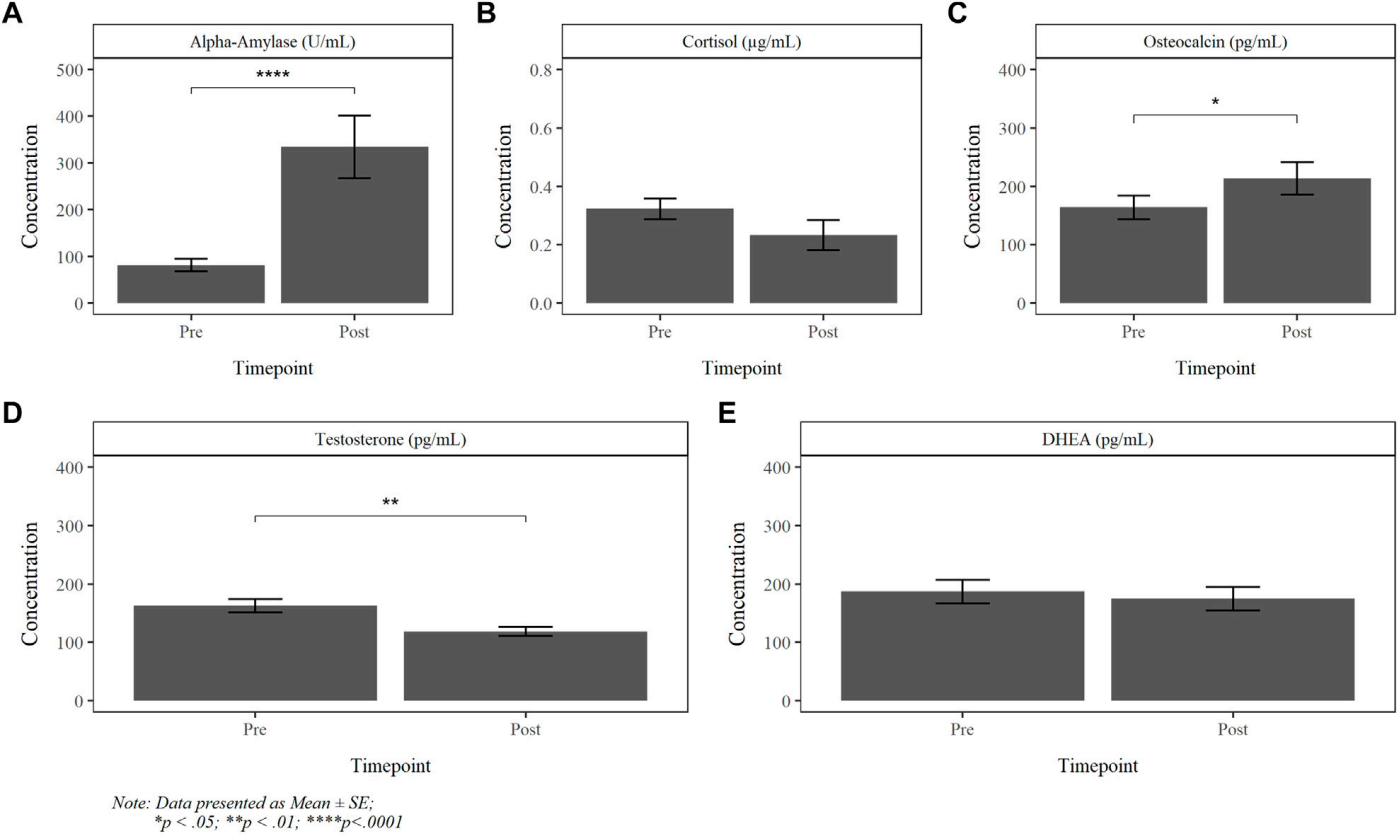
Absolute pre- and post-dive concentration of alpha-amylase **(A)**, cortisol **(B)**, osteocalcin **(C)**, testosterone **(D)** and DHEA **(E)**.

The absolute mean AA, CORT, OCN, TEST and DHEA salivary concentrations, obtained pre- and post-dive, are reported in Figure 5. There was a significant increase in AA, OCN and decrease in testosterone following the dive. Results revealed no statistically significant differences between dive groups. While cortisol exhibited divergent changes among PG1, PG2A and PG2B divers, the variance within the groups were too large to reach significance (*p* = 0.112).

### Correlation Analysis

#### Body size

Correlative analysis showed that there was no correlation between body surface area and change in core, mean skin, hand or foot temperatures.

#### Physiological metrics

There were no correlations between change in core, mean skin, hand and foot temperature and blood glucose, ketone, lactate and USG. Further, there were no relationship between any hormone and physiological metric.

#### Alpha-amylase

Further, there was no relationship between baseline (pre-dive) AA and any temperature metric. However, foot (*ρ* = -0.35, *p* = 0.18), hand (*ρ* = -0.42, *p* = 0.2) and mean skin temperature (*ρ* = -0.34, *p* = 0.20) exhibited weak to moderate non-statistically significant indirect relationships when comparing relative changes to AA and relative changes in temperature.

#### Cortisol

No relationship between pre-dive CORT and change in temperature were noted, yet there were moderate to strong trends between baseline cortisol concentrations and changes in foot temperature (*ρ* = 0.52, *p* = 0.071). However, a significant indirect correlation between changes in cortisol concentrations and changes in foot temperature (*ρ* = −0.5, *p* = 0.042) was detected and a similar relationship was noted with hand temperature, but it failed to reach statistical significance (*ρ* = −0.52, *p* = 0.104).

#### Osteocalcin

A significant direct correlation between baseline OCN and change in hand temperature (*ρ* = 0.66, *p* = 0.044) and significant indirect correlation between changes in OCN concentrations and changes in hand temperature (*ρ* = −0.59, *p* = 0.043) were noted. A strong non-significant inverse relationship comparing baseline OCN concentrations with changes in core temperature (*ρ* = −0.52, *p* = 0.107) was also observed.

#### Testosterone

No relationship between pre-dive TEST and change in temperature were noted yet, there were moderate to strong trends between baseline TEST concentrations and changes in core (*ρ* = −0.6, *p* = 0.056) and hand temperature (*ρ* = 0.62, *p* = 0.060).

#### DHEA

No relationship between DHEA and core, mean skin, hand and foot temperatures were noted. Correlations between the change in DHEA and OCN (*ρ* = 0.52, *p* = 0.046); DHEA and CORT (*ρ* = 0.65, *p* = 0.008); and DHEA and TEST (*ρ* = 0.7, *p* = 0.004) were noted.

## Discussion

The aim of this effort was to measure the acute stress response during prolonged extreme cold water diving and its potential influence on thermoregulation. The major findings of this study are that prolonged cold water diving increased salivary AA and OCN and decreased total TEST. Data suggests that changes in CORT and OCN are related to change in extremity temperature and that baseline values of these analytes may be indicative of thermoregulatory capacity in the extremities.

There is ample data on acute stress response in military personnel undergoing rigorous physical training and mental/psychological stress (Morgan et al., 2000b; Taylor, et al., 2007b; Castellani et al., 2017; Taylor et al., 2021). Different types of stressors led to varying effects on neuroendocrine responses; however, collectively, stress from interrogation, skydiving, and intense physical training resulted in elevated cortisol and DHEA and depressed testosterone (Morgan, et al., 2000a; Morgan et al., 2001; Morgan et al., 2004; Taylor MK. et al., 2007; Taylor, et al., 2007b; Morgan et al., 2009). Yet, to our knowledge there is no data on stress response to prolonged cold water exposure. Limited contradictory data exist in recreational SCUBA divers on HPA and SAM/SNSesponse to short duration dives (15–20 min) and in moderately cold water (15–19°C) (Weist et al., 2012; Zarezadeh & Azarbayjani, 2014; Marlinge et al., 2019). Findings from these efforts showed a mixed change in cortisol (e.g. increase, decrease or no change) along with a suppression of norepinephrine. The disparity between the current findings and those previously reported is likely due to the exposure duration. The acute rise of cortisol immediately following submersion that has been previously reported may have occurred in the current effort; however, as duration of submersion and cold exposure persisted, negative feedback inhibition of continued cortisol secretion likely attenuated the response (De Kloet et al., 1998; Shida et al., 2020)—except in individuals under perceived physiological stress. The inverse relationship between CORT and foot temperature indicated a heightened stress response in individuals nearing dive termination criteria of skin temperatures of 10°C. A similar, albeit, non-significant pattern was noticed in individuals with cold hands. The stress-induced increase in cortisol is to prepare for “fight-or-flight”; to increase glucose or energy supply to the body as well as mediate systemic changes in arousal and maintain homeostasis (Lee et al., 2012). Thus, the decreased extremity temperature likely provoked an extended CORT response which was more pronounced in PG1 divers who were diving at a greater depth. Zarezadeh and Azarbayjani (2014) found submersion depth influences CORT at depths starting at 32 fsw in warm water (24°C). While the depth in the current study was less than 32 fsw, the time under hyperbaria (9 h vs 20 min) and water temperature (4°C vs 24°C) was vastly different, which may explain the variance in findings.

Coupled to CORT release is secretion of DHEA. Both hormones are secreted in response to HPA activation and normally increase in response to physical and psychological stress (Kamin & Kertes, 2017). However, long term stress exposure (Lennartsson et al., 2013) and training stress (Bouget et al., 2006) has been shown to attenuate DHEA levels. Both CORT and DHEA decreased non-significantly over time but were strongly correlated. Due to the duration of the dive, diurnal influence may have affected these biomarkers as both normally peak in the morning and reach a nadir by the late afternoon. Alternatively, vagal inhibition of the HPA axis in response to cold to conserve energy and oxygen via reduction in heart rate could be a factor (Baranova et al., 2010; Godek and Freeman, 2021). All participants were experienced military divers and while the initial CSR may have been invoked, rapid adaptation likely attenuated the stress response (Grissom & Bhatnagar, 2009), except in those individuals reaching or approaching the extremity skin temperature threshold of 10°C.

In parallel with HPA axis, the more rapid SAM/SNS response can be measured *via* AA, a known surrogate marker of norepinephrine/epinephrine (NE/Epi) (Chrousos and Gold, 1992; Granger et al., 2007; Nater and Rohleder, 2009). In the current study, AA increased between 200-225% following the prolonged cold water exposure. Cold stress stimulates vasoconstriction at the periphery in order to shunt blood to the core and deep tissues, vital for survival. This response is mediated via *α*-adrenergic receptor activation driven by an increase in NE/Epi upon cold exposure. Peripheral skin and extremity temperature significantly decreased over time. While not statistically significant, there were moderately strong correlations to the percent change in peripheral foot and hand temperature and percent change in AA. Due to this study folding into active training, glove and bootie configurations were not controlled for and thus, the variation in percent change in mean skin temperature and AA response might be confounded by protective equipment. Nevertheless, the inverse trend indicates a relationship between thermoregulation and AA secretion and warrants further investigation. Exercise elicits a substantial intensity-dependent increase in AA (Granger et al., 2007; Koibuchi and Suzuki, 2014). However, the current study had little to no activity and thus, the substantive increase in AA cannot be attributed to physical exertion. Mental stress can provoke a pronounced SAM/SNS response and the prolonged cold exposure coupled to the lack of caloric intake may have exacerbated the AA response in order to mobilize fuel source and maintain homeostasis. Blood glucose levels, though decreased, remained within normal physiological range over 9 h despite not consuming any food and there was a slight increase in ketones indicative of increased lipid oxidation (Cahill et al., 1966).

OCN is a hormone secreted from bone in response to metabolic stress and functions to maintain glucose homeostasis (Wei and Karsenty, 2015; Diaz-Franco et al., 2019). This bone derived marker has been implicated as a potential stress biomarker secreted in response to exercise (Mohammad Rahimi et al., 2021) and functions to block parasympathetic activation in order to maintain sympathetic tone (Berger et al., 2019). OCN significantly increased over time and was inversely related to hand temperature in the current effort. Further, OCN was related to DHEA. While correlative and not causative, both metrics suggest prolonged HPA/SNS activation (stress response]. Vasoconstriction at the periphery is regulated by *α*-adrenergic input and the concomitant rise in AA supports the interaction of these endocrine pathways and potential role in thermoregulation during prolonged cold stress. While, not statistically significant, it is worth noting that there was variable increase in OCN among the dive groups (PG2A: 15.34%, PG2B: 9.65%; PG1:49.3%) suggesting that pressure (depth) may affect secretion. PG1 divers were submerged the entire 9 h, whereas PG2A and PG2B had 5 h at or near the surface. Hyperbaric oxygen therapy increases bone remodeling (Chiu et al., 2020); thus, the substantial increase in PG1 may be due to time at depth and the gas mixture being used. To our knowledge, this is the first study to measure OCN during submersion or immersion in humans and more work is needed in order to determine hyperbaric regulation of OCN secretion and subsequent role in thermoregulation. Moreover, OCN modulates energy expenditure in muscle, increasing lipid oxidation and expression of genes involved in thermogenesis (Wolf, 2008; Ferron and Lacombe, 2014) which may further affect thermoregulatory capacity. However, in this effort, we did not see any correlation between blood fuel changes and OCN or impact on thermoregulation. This maybe that OCN regulation occurs at the muscular level and has no impact on circulating glucose levels (Lin et al., 2018; Starup-Linde et al., 2018). Alternatively, all participants were experienced divers and despite the prolonged duration in cold-water, they exhibited homeostatic regulation.

All divers were experienced and thus, the acute stress response likely can be attributed to the extreme cold water and not to mental stress of diving. Duration of exposure and the impact on metabolism to maintain thermoregulation could have influenced stress response, as we recently reported an increase in metabolism of 53% during a 6-h dive in similar water temperatures (Chapin et al., 2021). However, to our knowledge there are no studies that have measured the impact of acute stress on metabolism and thermoregulation while diving. There are some limitations to this effort that need to be addressed. There were no dietary, caffeine or nicotine restrictions. All pre-dive hormones were measured prior to eating and post-dive metrics are reflective of a “fast” as divers had not consumed food or water for 9 h while under water. While circadian influence on body temperature is critical during exercise, it is not as applicable in this effort as divers were passive for the duration of the dive (Ravanelli & Jay, 2021). All biological samples were acquired approximately 2 h after wake and thus baseline values were likely not influenced by an awakening response and were a reflection of the stress of cold training even with the prolonged dive approaching natural nadir of CORT and TEST (Kudielka et al., 2004).

Despite the limitations, these data suggest that long duration, cold water diving initiates a stress response and that peripheral extremity temperature (e.g., hands and feet) may alter the HPA axis responses during long duration, cold water diving or vice versa. These data are limited and more work is needed to fully understand the impact of repetitive cold water exposure on the stress response especially with respect to thermoregulation and metabolism.

## Data Availability

The raw data supporting the conclusions of this article will be made available by the authors, without undue reservation.
